# Review and Evaluation of European National Clinical Practice Guidelines for the Treatment and Management of Active Charcot Neuro-Osteoarthropathy in Diabetes Using the AGREE-II Tool Identifies an Absence of Evidence-Based Recommendations

**DOI:** 10.1155/2024/7533891

**Published:** 2024-06-10

**Authors:** Nichola Renwick, Jennifer Pallin, Rasmus Bo Jansen, Catherine Gooday, Aroa Tardáguila-Garcia, Irene Sanz-Corbalán, Anastasios Tentolouris, Alexandra Jirkovská, Armin Koller, Anna Korzon-Burakowska, Nina Petrova, Frances Game

**Affiliations:** ^1^ School of Sports, Health and Exercise Science University of Portsmouth, Portsmouth, UK; ^2^ School of Public Health University College Cork, Cork, Ireland; ^3^ Bispebjerg Hospital University of Copenhagen, Copenhagen, Denmark; ^4^ Elsie Bertram Diabetes Centre Norfolk & Norwich University Hospitals NHS Foundation Trust, Norwich, UK; ^5^ Diabetic Foot Unit Complutense University of Madrid, Madrid, Spain; ^6^ School of Medicine National and Kapodistrian University of Athens, Athens, Greece; ^7^ Diabetes Centre Institute for Clinical and Experimental Medicine, Prague, Czech Republic; ^8^ Technical Orthopaedics & Diabetic Foot Surgery Klinik Dr. Guth, Hamburg, Germany; ^9^ Division of Preventive Medicine & Education Medical University of Gdańsk, Gdańsk, Poland; ^10^ Diabetic Foot Clinic King's College Hospital NHS Foundation Trust, London, UK; ^11^ Department of Diabetes and Endocrinology University Hospitals of Derby and Burton NHS Foundation Trust, Derby, UK

**Keywords:** Charcot foot, Charcot neuro-osteoarthropathy, Clinical guideline, Diabetic foot, European guidelines

## Abstract

**Background:** Charcot neuro-osteoarthropathy (CNO) is a rare but devastating complication of diabetes associated with high rates of morbidity; yet, many nonfoot specialists are unaware of it, resulting in missed and delayed diagnosis. Clinical practice guidelines (CPGs) have proven useful in improving quality of care and standardizing practice in diabetes and diabetic foot care. However, little is known about the consistency in recommendations for identification and management of active CNO.

**Aim:** The aim of this study is to review European national diabetes CPGs for the diagnosis and management of active CNO and to assess their methodological rigor and transparency.

**Methods:** A systematic search was performed to identify diabetes national CPGs across Europe. Guidelines in any language were reviewed to explore whether they provided a definition for active CNO and recommendations for diagnosis, monitoring, and management. Methodological rigor and transparency were assessed using the Appraisal of Guidelines for Research and Evaluation (AGREE-II) tool, which comprises 23 key items organized within six domains with an overall guideline assessment score of ≥ 60% considered to be of adequate quality to recommend use. Each guideline was assessed by two reviewers, and inter-rater agreement (Kendall's *W*) was calculated for AGREE-II scores.

**Results:** Seventeen CPGs met the inclusion criteria. Breadth of CNO content varied across guidelines (median (IQR) word count: 327; Q1 = 151; Q3 = 790), and 53% provided a definition for active CNO. Recommendations for diagnosis and monitoring were provided by 82% and 53%, respectively, with offloading being the most common management recommendation (88%). Four guidelines (24%) reached threshold for recommendation for use in clinical practice (≥ 60%) with the scope and purpose domain scoring highest (mean (SD): 67%, ± 23%). The remaining domains had average scores ranging between 19% and 53%. Inter-rater agreement was strong (*W* = 0.882; *p* < 0.001).

**Conclusions:** European national CPGs for diabetes provide limited recommendations on active CNO. All guidelines showcased deficits in their methodology, suggesting that more rigorous methods should be employed for diabetes CPG development across Europe.

## 1. Introduction

Charcot neuro-osteoarthropathy (CNO), also known as the Charcot foot, is an inflammatory process affecting the bones, joints, and soft tissues of the foot and ankle in persons with peripheral neuropathy [1]. Although it most commonly occurs in people with diabetes, it can materialize as a result of any condition in which peripheral neuropathy occurs [2]. It is an acute localized inflammatory condition which can lead to bone destruction, subluxation, dislocation, and deformity in the absence of pain, which in turn can cause permanent deformity of the foot, increasing a person's risk of developing ulcerations and undergoing amputations [3, 4]. Furthermore, CNO is associated with significant negative impacts on the quality of life, impacting a person's ability to work and socialize, leading to feelings of isolation [5–7].

Despite its significant impact on morbidity, the incidence and prevalence of CNO are relatively unknown, with the estimated prevalence of CNO being 0.56% in people with diabetes, increasing to 10%–12% for those in tertiary settings [8–10]. However, evidence suggests that incidence and prevalence of CNO are often under-reported due to misdiagnosis by healthcare professionals [11, 12]. In addition, diagnosis of active CNO is often delayed, with a recent systematic review showing that 53.2% of CNO cases experience a delay in diagnosis (95% CI: 28.9%–77.4%), with a median delay of approximately 87 days (95% CI: 10.5–162.1) [11]. This is of concern as delays in managing the active Charcot foot have shown to be associated with worse outcomes, including increased foot deformity and higher incidence of ulceration and amputation, which are independently associated with increased morbidity and mortality [6, 11, 13–16].

One reason for delayed, and missed, diagnosis may be because clinical signs and symptoms, including redness, heat, and swelling, are often mistaken for other common conditions such as osteomyelitis, deep vein thrombosis, arthritis, cellulitis, or ankle sprains [11, 17]. One study found that, in a survey of nonfoot specialists, 68% reported having poor or no knowledge of CNO [18]. If clinicians are not suspicious of active CNO, then those affected are unlikely to receive plain radiographs and/or magnetic resonance imaging (MRI) of the affected limb and “gold standard” limb immobilization, in line with the best practice evidence. Another reported reason for potential delays in diagnosis is because of unclear pathways and protocols within health systems and confusion around types of effective tools for diagnosing and monitoring active CNO [17].

One way of standardizing disease diagnosis and management, and improving quality of care, is through evidence-based clinical practice guidelines (CPGs) [19, 20]. High-quality CPGs can enable clinicians to make evidence-based decisions in a time-efficient manner, while also reducing practice discrepancies across professions and settings ensuring improved healthcare. More organizations and health systems are publishing their own CPGs, with different organizations often providing conflicting recommendations. We have seen this internationally with diabetic foot guidelines [21, 22]. However, little is known about the consistency in recommendations for identification and management of active CNO. This is of concern as conflicting recommendations can result in confusion and raises concerns about the quality of CPGs and the underlying evidence [23]. To improve identification and management of active CNO, identifying CNO diagnosis and management recommendations within guidelines and assessing the quality of guidelines is essential. Therefore, the primary aim of this study was to identify European national diabetes and diabetic foot CPGs and assess whether they provided recommendations on treatment and management of CNO. Secondary aims included (1) to explore whether identified guidelines provide a definition for active CNO and remission, as well as advice on diagnosis and monitoring, and (2) to assess the methodological rigor and transparency of identified guidelines using the Appraisal of Guidelines for Research and Evaluation (AGREE) tool which has been developed to assess the methodological quality of CPGs [24].

## 2. Materials and Methods

This scoping review was performed in line with recommendations outlined by the Joanna Briggs Institute (JBI) and is reported in accordance with the Preferred Reporting Items for Systematic reviews and Meta-Analyses extension for Scoping Reviews (PRISMA-ScR) guidelines, the checklist of which is outlined in Supporting Information 1 [25–27].

### 2.1. Search Strategy

We used multiple methods to identify relevant CPGs. First, we performed a systematic search on March 16, 2022, in PubMed, ScienceDirect, Scopus, and CINAHL databases, using the following search strategy: core search: “Charcot Neur∗”[Title/Abstract] OR “Diabetic Foot∗” OR “Charcot Arthro∗” OR “Charcot Foot∗” OR Osteoarthropathy OR “Charcot joint”.

It is combined with Boolean operator AND:

1. (guid∗∗[All Fields])
2. (recommendation∗)
3. (treatment∗ OR management∗ OR diagnosis∗)[All Fields] (assess∗ OR monitor∗)

This search strategy was developed in consultation with a research librarian. We used search terms for CPGs, diabetes, foot diabetes, and Charcot neuroarthropathy. Second, reference lists of the selected studies were searched to identify any additional relevant CPGs. Third, we conducted a web search of “Google” and “Google Scholar” using the same search terms. Fourth, we sent a letter (see Supporting Information 2) to the members of the D-Foot International network [28] requesting copies of relevant CPGs. Fifth, the authors used their professional contacts to identify guidelines. Finally, at the annual Diabetic Foot Study Group (DFSG) meeting held in Bratislava, Slovenia, in September 2022, the authors informed attendees of what countries they were missing and asked attendees to email the authors with guidelines for their relevant countries. At the same conference, the letters circulated to the D-Foot International network were also provided to attendees.

### 2.2. Inclusion and Exclusion Criteria

We included national diabetes or diabetic foot CPGs that met the Institute of Medicine definition of a CPG [29], published in or after the year 2000 until December 2022, from countries within the European continent. There were no limitations on language. We excluded controlled trials, observational studies, qualitative studies, and narrative reviews. Only the latest version of a guideline was selected for inclusion within the review.

### 2.3. Study Selection

We downloaded all the papers into Rayyan (http://rayyan.qcri.org) and removed duplicates. Two reviewers (A.T. and R.B.J.) independently conducted screening. Where consensus could not be met, a third reviewer (C.G.) was consulted. CPGs were identified through D-Foot International, and the authors' networks were screened against the eligibility criteria by the same reviewers. All records deemed eligible following this consensus process were included for assessment and data extraction. Seven independent reviewers were involved in data extraction and assessment of the guidelines (J.P., C.G., N.R., A.T.-G., A.T., I.S.-C., and R.B.J.). If a reviewer was involved in the development of a guideline, then they were excluded from the scoring process.

### 2.4. Translating Guidelines

Guidelines were translated to the reviewer's native language using DEEPL or Google Translate. For example, reviewers from Spain translated guidelines to Spanish, and reviewers from the United Kingdom and Ireland translated guidelines to English.

### 2.5. Data Extraction and Synthesis

Two people independently extracted data from each CPG using a prespecified form (see Supporting Information 3 for data extraction items). We extracted information on guideline native language, authors (institution/organization), year of publication, and focus of the guideline (e.g., did it focus on general diabetes or diabetic foot specifically). Furthermore, to assess similarities and disparities between CPGs, we extracted data to identify whether the following components were included within guidelines: definition for active CNO and remission, recommendation on diagnosis, monitoring, and management (including offloading and pharmacological and surgical treatment) of active CNO. We did not compare specific definitions and recommendations within guidelines; we only assessed whether they were included within the guidelines. After guidelines were translated into English, the number of words on CNO within guidelines was counted.

A narrative synthesis approach was used to summarize information relating to definitions and recommendations provided. This was used as it allows for review and synthesis of findings from multiple studies, relying primarily on the use of words and text to summarize and explain the findings [30].

### 2.6. AGREE-II Assessment

We did not seek to assess the quality and evidence base behind the specific recommendations. However, we evaluated the methodological rigor and transparency of each CPG using the AGREE-II instrument [9]. This instrument consists of 23 items organized into 6 domains: scope and purpose (Domain 1), stakeholder involvement (Domain 2), rigor of development (Domain 3), clarity of presentation (Domain 4), applicability (Domain 5), and editorial independence (Domain 6). Only the Charcot component was assessed within Domains 4 and 5, whereas for the remaining domains, the whole guideline was assessed. An extensive list of the domains and items can be found in Supporting Information 4.

Two reviewers independently scored the CPG against each statement using a Likert-type scale ranging from 1 (*strongly disagree*) to 7 (*strongly agree*). We used the methodology in the AGREE-II instrument manual [24] to calculate the domain score expressed as a percentage.

Strength of domain scores was determined from previously established thresholds, whereby a score of 60% or above was considered as being effectively addressed [31–34]. Guidelines were classed as recommended and of high quality if they scored above 60% in three or more domains [24].

### 2.7. Inter-Rater Agreement

Following a Shapiro-Wilk test which indicated non-normal distribution, nonparametric statistics were used. Kendall's coefficient of concordance (*W*) was used to determine inter-rater agreement between all assessors across all 17 guidelines.

## 3. Results

### 3.1. Search Results

**Figure 1:**
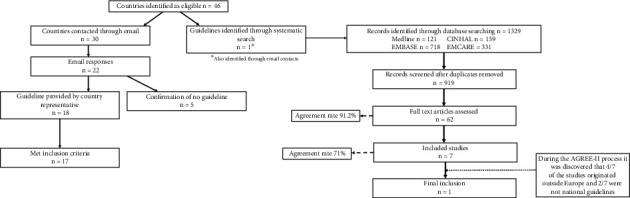
PRISMA flow diagram summarizing how guidelines were identified and obtained.

Out of the 46 countries identified as eligible, we were able to contact representatives from 30, and of these, 22 (73%) responded. Representatives from five countries advised us that they did not have a relevant guideline and eight countries did not respond to our request for information. The systematic search identified one guideline which met the inclusion criteria. In total, 17 guidelines were identified and assessed (Figure 1). All guidelines assessed were published between 2004 and 2021. Each guideline was reviewed by two reviewers, and inter-rater agreement was considered strong (*W* = 0.882, *p* < 0.001). A list of eligible countries, their responses, and reviewers assigned to assess each guideline can be found in Supporting Information 5.

### 3.2. Guideline Content

Most guidelines (*n* = 14) focused on management of the foot in diabetes. The median (IQR) word count for information relating to CNO was 327 (Q1 = 151, Q3 = 790). As outlined in Table 1, a definition for active CNO was recorded in 53% (*n* = 9) of guidelines, while 82% (*n* = 14) provided recommendations on diagnosing active CNO and 53% (*n* = 9) provided recommendations on monitoring the active Charcot foot. Regarding management, offloading was the most common recommendation provided in 88% (*n* = 15) followed by recommendations on pharmacological treatments in 47% (*n* = 8) and surgical treatment in 53% (*n* = 9). A definition for remission of the active Charcot foot was provided in 35% (*n* = 6) of guidelines, and recommendations for long-term follow-up were discussed in 29% (*n* = 5).

### 3.3. Guideline Quality Rated Using the AGREE-II Tool

As outlined in Table 2, following assessment against the AGREE-II tool, 24% (*n* = 4) of guidelines [35–38] scored ≥ 60% in three or more domains meaning they could be recommended for use in clinical practice. The overall highest scoring guideline was from England, Wales, and Northern Ireland which scored ≥ 60% in five out of six domains [35]. The scope and purpose domain received the highest score on average (mean 67%, SD 23%) while the remaining five domains had an average score range of 19%–53%.

#### 3.3.1. Scope and Purpose

The scope and purpose domain evaluated the overall objectives, health questions, and target population of the guideline. This domain had the highest average score, 67% (SD 23%); however, individual scores ranged from 0% to 100%. The threshold for being considered effectively addressed was achieved for 12 out of 17 guidelines (71%). One guideline [39] scored 0% as it failed to provide any specific description of the objectives, health questions, and target population of the guideline.

#### 3.3.2. Stakeholder Involvement

This domain seeks to assess which professional groups are involved in the development of the guideline, if the target population of the guideline is involved in the development of the guideline and if the target users of the guidelines are clearly defined. The average score for this domain was 37% (SD 29%) with 24% (*n* = 4) of the guidelines reaching the threshold for effectively addressing these points. Five guidelines (29%) did provide a description of which professional groups were involved in the guideline development [40–43]. Two guidelines were identified as including the views of the target population in the development of the guideline [36, 37]. Six guidelines (35%) failed to identify who the target users of the guideline were [39–41, 43–45].

#### 3.3.3. Rigor of Development

This domain looked to assess the methodological rigor for the development of the guideline across eight items. The average score for this domain was 34% (SD 32%) with 24% (*n* = 4) of guidelines reaching the threshold for being considered effectively addressed. Systematic methods for collecting evidence for the guidelines were described by six [35, 36, 38, 40, 43, 46]. Five guidelines (29%) clearly demonstrated the link between the recommendations and evidence [35, 36, 38, 42, 43]. Four guidelines (24%) clearly described the strengths and limitation of the body of evidence [35, 40, 43, 46]. Five guidelines (29%) clearly described the methodology behind the formation of the recommendations [35, 36, 40, 43, 46]. Only two guidelines demonstrated clearly that they considered the health benefits, risks, and side effects when forming the guidelines [35, 43]. Ten guidelines (59%) did not provide or mention any external review prior to publication [37–39, 41–45, 47, 48]. Five guidelines (29%) provided a procedure for updating the recommendations [35–37, 46, 47].

#### 3.3.4. Clarity of Presentation (Charcot-Related Content Only)

This domain looks to assess whether the recommendations are specific and easily identifiable and if different options for management of the condition are presented. This domain scored an average of 53% (SD 29%), and six guidelines reached the threshold for effectively addressing the domain [35, 37, 38, 42, 47]. One guideline failed to provide clear and unambiguous recommendations, did not provide different management options, and failed to clearly identify the key recommendations [42].

#### 3.3.5. Applicability (Charcot-Related Content Only)

This domain seeks to assess the applicability of the guideline through criteria on facilitators and barriers, application to clinical practice, resource implications for implementation of recommendations, and auditing of the recommendations. This domain had the lowest average score of 19% (SD 17%) with none of the guidelines assessed reaching the threshold for effectively addressing the domain. Scores for this domain ranged from 0% to 52%.

#### 3.3.6. Editorial Independence

This domain evaluates the interests of the funding body and any conflicts of interest from the guideline development group. The average score for this domain was 29% (SD 29%) with three guidelines (18%) reaching the threshold for effectively addressing the items [35, 37, 45]. Only one guideline [35] provided clear information on whether the views of the funding body had influence on the guideline and six guidelines provided clearly disclosed any competing interests of the guideline development group [37–39, 42, 46, 48].

## 4. Discussion

This review sought to identify European national diabetes and diabetic foot CPGs and assess whether they provided recommendations on treatment and management of CNO. We also explored whether they provided a definition for active CNO and remission, as well as advice on diagnosis and monitoring, while also assessing their methodological rigor and transparency using an internationally recognized tool.

Out of the 46 eligible countries, we were only able to identify 17 CPGs that were eligible for inclusion in this study and received confirmation of no guideline for five countries (16%). Despite our thorough search strategy, CPGs were not readily accessible. Perhaps, guidelines do exist, but our review suggests that many may not be readily available to clinicians. In many countries, guidelines are often developed by organizations outside of health systems, meaning guidelines may not be embedded into healthcare. Diabetic foot disease is a devastating complication of diabetes, with internationally recognized prevention and management strategies [49]. However, results from our review also suggest that this evidence is not being translated into CPGs, which have been identified as playing a key role in supporting clinicians and improving healthcare [19, 20]. While the IWGDF has recently published an international guideline created by an international consortium of clinical and academic experts who specialize in CNO for the diagnosis and treatment of active CNO [51], this may not translate into inclusion into national CPGs [22]. The identified lack of diabetic foot guidelines in our review is of concern as it suggests a lack of support for clinicians to identify and manage diabetic foot disease. Furthermore, where there is a diabetic foot guideline, little attention is given to CNO, despite its significant impact on morbidity and mortality [5, 7, 50]. We found that the level to which CNO was discussed varied substantially across guidelines as illustrated by the word count for information relating to CNO (median (IQR): 327 (Q1 = 151, Q3 = 790)). This suggests that identification and management of CNO provided little focus within CPGs, which may be why clinicians are often not aware of it as a diabetic foot complication or why there is delay in identifying active CNO [11, 17, 18].

Within the CPGs identified, only 50% provided a definition for active CNO, with fewer (33%) providing a definition for the foot in remission. This is of concern because we know that consistency in terminology is important in diabetes care and education and ensuring clear clinical communication [51]. Identification and management of active CNO is already a clinical issue, with many nonfoot specialists being unaware of it and many people experiencing delayed diagnosis [18]. This review identified that the inclusion of a definition of CNO varies between guidelines which could potentially add to this confusion.

While most guidelines provided recommendations on diagnosing active CNO, few provided recommendations on monitoring the active Charcot foot. This may be reflective of the fact that up until recently, there has been no internationally agreed recommendations on diagnosis and management of CNO and the recognized uncertainty around the effectiveness of monitoring techniques of CNO [5, 52], and so national guidelines may have been unable to provide clear recommendations based on evidence. However, some did provide recommendation on management with offloading and immobilization of the affected foot being most recommended (83%), followed by pharmacological (50%) and surgical (44%) treatment recommendations.

Regarding the methodological rigor and transparency of identified guidelines, we found that no guideline achieved a score greater than 60% in all domains, meaning all guidelines had shortfalls that should be addressed in future publications. As we were primarily interested in the evidence surrounding active CNO, the whole document was assessed for Domains 1, 2, 3, and 6, whereas only the CNO content was assessed for Domains 4 and 5 allowing us to assess the clarity of presentation and applicability of recommendations for active CNO. Overall, we found a lack of clarity in recommendations, with only six guidelines achieving a score greater than 60% [35, 37, 38, 45, 47, 48]. These low scores are of concern, as lack of clarity in the presentation (i.e., language used and formatting) can have a direct impact on the usability of the guideline to the end users. Hard-to-read texts are often unusable in a busy, clinical setting, and evidence suggests that CPGs are less likely to be followed if they contained controversial or vague recommendations [23]. Failure to differentiate the most important parts of a guideline can also become time-consuming to the clinicians and patients. Regarding Domain 6 (applicability), this was the poorest performing domain (20 ± 18), with no guideline achieving threshold for recommendation suggesting that users are not being guided on how to implement recommendations. This is not an isolated issue though, as it has been the poorest performing domain in other studies assessing the methodological quality of diabetic foot related CPGs [31, 53] and other CPGs internationally [54]. Implementation strategies have been identified as being essential to encourage CPG uptake among relevant stakeholders; however, despite a lack of implementation of guidance, it is unclear whether current recommendations within CPGs are being put into practice. A lack of implementation may be a contributor to the widely acknowledged delay in diagnosis and treatment for CNO as clinicians may not be aware of recommendations within the relevant CPG.

Regarding the whole guidelines, Domain 1 (scope and practice) achieved the highest score overall, which is consistent with others who have evaluated diabetic foot guidelines [21, 53]. However, six guidelines still scored below threshold and one guideline received a score of zero, suggesting that some guideline developers are not clearly outlining the overall aim of the guideline, the specific health questions, and the target population. It is well established that a clear sense of the target audience informs subsequent decisions about the guideline's scope, objectives, and format and style of wording which may explain why those who scored poorly in Domain 1 also scored poorly in the subsequent domains [55]. Our results also suggest a lack of stakeholder engagement, with thirteen guidelines failing to reach threshold. This is of concern as the importance of stakeholder engagement in CPG development is seen an important consideration to ensure guideline's acceptability and feasibility to the end users, and topics that are of priority are being incorporated [56]. Failure to focus on the topics important to patients, and end users, might lead to the guideline being less well received, low adherence from both groups, and not being implemented fully. Domain 3 (rigor of development) also scored quite poorly receiving an overall score of 35 ± 33, with thirteen guidelines failing to reach threshold suggesting that they failed to provide evidence that systematic methods were used to formulate guideline recommendations. This could mean that either the evidence is sparse, the guideline authors have missed available evidence, or they may be biased for/against certain practices. Either way, a low score in this domain makes it harder for the end user to work out why certain topics are underrepresented in the material and again may contribute to poor diagnosis and treatment plans for CNO.

Nonetheless, all CPGs identified during this review showcased deficits in their methodology when assessed against the AGREE-II criteria; perhaps, groups involved in CPG development should incorporate appropriate tools that are designed to enhance CPG development and ensure an evidence-based robust methodology with unambiguous recommendations. This would help clinicians be more knowledgeable and confident in diagnosing and managing CNO.

### 4.1. Strengths and Limitations

To our knowledge, this is the first study to evaluate diabetic foot guidelines across Europe, with a focus on recommendations on definitions and recommendations for active CNO diagnosis and management. In addition, we were able to provide a comprehensive picture of the guidelines as we placed no limitations on language; while we acknowledge that our methods of language translation could be more standardized to reduce language biases [57], our inter-rater agreement was strong (*W* = 0.882, *p* < 0.001) which suggests consistent data extraction and interpretation. This study had further limitations. First, because CNO most commonly occurs in people with diabetes [1], and those affected most likely to be managed within a diabetic foot multidisciplinary team, the search strategy was limited to only include diabetes and diabetic foot guidelines. As a result, some guidelines may have been missed as they sit within other specialities which may account for the low yield of guidelines obtained in this study (41%). For example, in Ireland, recommendations for CNO sit within wound care guidelines but because it was not a specific diabetes-related CPG and it was not eligible for inclusion [58] Second, we assessed whether guidelines contained definitions and recommendations, but we did not assess the evidence base behind the recommendations meaning that while guidelines may have been providing recommendations, the evidence base to support these may have been poor. Third, while the AGREE-II tool is the most used guideline appraisal tool, it might not be well suited to evaluate the quality of what constitutes a “good” guideline for every type of healthcare system. It is possible that the domains that AGREE-II focuses on are not in line with what every country needs or what their healthcare traditions are. This could be the reason why a lot of the guidelines “fail” in certain areas such as rigor of development and/or applicability. Thus, a low AGREE-II score does not necessarily indicate a poor methodological approach to guideline development but that a clear description for the developmental process is needed. In addition, it is important to underline that the AGREE-II tool does not say anything about the quality of the contents of a guideline. Thus, a guideline that scores low can still contain accurate information and be useful for healthcare professionals.

To summarize, this study found that information on CNO within national European CPGs was limited or absent. In addition, when assessed against the AGREE-II criteria, all guidelines showcased deficits in their methodology, and perhaps, groups involved in CPG development should incorporate appropriate tools that are designed to enhance development and implementation and ensure an evidence-based robust methodology with unambiguous recommendations. Given the uncertainties that exist for the diagnosis and management of CNO, there is a need for clear evidence-based guidelines. Further work is needed to determine if CPG recommendations are being implemented in practice and what care and management are provided for people with CNO when no guideline is available.

## Figures and Tables

**Table 1:** Summary results of scoping review identifying focus area and Charcot neuro-osteoarthropathy content from national guidelines.

**Country**	**Focus area of guideline**			**General assessment of CN**			**Management of CN**			**Remission of CN**			
	**Diabetes**	**Diabetic foot**	**Surgery**	**Definition**	**Diagnosis**	**Monitoring**	**Offloading**	**Pharmacology**	**Surgery**	**Definition**	**Recommendation for follow-up**		**Word count for CN content**
Austria [48]	**x**	**✓**	**x**	**x**	**x**	**x**	**✓**	**x**	**x**	**x**	**x**		151
Czechia [42]	**x**	**✓**	**✓**	**✓**	**✓**	**✓**	**✓**	**x**	**✓**	**✓**	**✓**		790
Denmark [41]	**x**	**✓**	**✓**	**✓**	**✓**	**✓**	**✓**	**✓**	**✓**	**✓**	**x**		793
England, Wales, and Northern Ireland [35]	**x**	**✓**	**x**	**x**	**✓**	**✓**	**✓**	**✓**	**x**	**✓**	**✓**		365
Finland [38]	**x**	**✓**	**✓**	**✓**	**✓**	**✓**	**✓**	**✓**	**✓**	**✓**	**✓**		1536
Germany [39]	**x**	**✓**	**x**	**x**	**✓**	**x**	**✓**	**x**	**x**	**x**	**x**		279
Greece [45]	**✓**	**x**	**x**	**✓**	**✓**	**x**	**✓**	**x**	**✓**	**x**	**x**		240
Kazakhstan [46]	**x**	**✓**	**x**	**x**	**✓**	**x**	**✓**	**x**	**x**	**x**	**x**		116
Netherlands [43]	**x**	**✓**	**✓**	**✓**	**✓**	**✓**	**✓**	**✓**	**✓**	**✓**	**✓**		7486
Poland [47]	**✓**	**x**	**x**	**x**	**✓**	**x**	**✓**	**✓**	**✓**	**x**	**x**		177
Romania [59]	**✓**	**✓**	**x**	**x**	**x**	**x**	**x**	**x**	**x**	**x**	**x**		60
Russia [40]	**x**	**✓**	**✓**	**✓**	**✓**	**✓**	**✓**	**✓**	**✓**	**✓**	**x**		1642
Scotland [36]	**✓**	**x**	**x**	**✓**	**✓**	**✓**	**✓**	**✓**	**x**	**x**	**x**		327
Slovenia [60]	**✓**	**✓**	**x**	**x**	**x**	**x**	**x**	**x**	**x**	**x**	**x**		13
Spain [44]	**x**	**✓**	**x**	**✓**	**✓**	**✓**	**✓**	**x**	**✓**	**x**	**x**		640
Sweden [61]	**x**	**✓**	**x**	**x**	**✓**	**x**	**✓**	**x**	**x**	**x**	**x**		125
Switzerland [37]	**x**	**✓**	**x**	**✓**	**✓**	**✓**	**✓**	**✓**	**✓**	**✓**	**✓**		765
Total (out of 17)	4	15	5	9	14	9	15	8	9	6	5	Median	327
% total	24	88	29	53	82	53	88	47	53	35	29	IQR	Q1 = 151, Q3 = 790

**Table 2:** Domain scores (%) for each national guideline with mean domain score (%) and standard deviation. Overall assessment of national guidelines is provided which identifies the number of domains reaching the threshold for being considered effectively addressed (≥ 60%) and their recommendation for use in clinical practice.

**Country**	**Domain 1**	**Domain 2**	**Domain 3**	**Domain 4**	**Domain 5**	**Domain 6**	**Overall assessment**	
	**Scope and purpose**	**Stakeholder involvement**	**Rigor of development**	**Clarity of presentation**	**Applicability**	**Editorial independence**	**Number of domains ≥ 60%**	**Recommended for clinical practice**
Austria [48]	86	19	4	50	2	50	1	No
Czechia [42]	44	28	23	89	29	0	1	No
Denmark [41]	67	0	20	58	15	0	1	No
England, Wales, and Northern Ireland [35]	81	86	98	89	29	75	**5**	**Yes**
Finland [38]	75	67	44	78	40	50	**3**	**Yes**
Germany [39]	0	14	0	33	0	50	0	No
Greece [45]	75	25	2	28	13	63	2	No
Kazakhstan [46]	67	58	72	28	6	50	2	No
Netherlands [43]	56	3	73	89	42	0	2	No
Poland [47]	94	42	19	75	2	33	2	No
Romania [59]	69	19	0	0	0	0	1	No
Russia [40]	56	11	59	53	27	4	0	No
Scotland [36]	100	97	72	58	23	42	**3**	**Yes**
Slovenia [60]	69	47	22	6	0	0	0	No
Spain [44]	56	8	4	58	2	0	0	No
Sweden [61]	64	44	8	22	33	4	1	No
Switzerland [37]	81	64	53	86	52>	71	**4**	**Yes**
Mean (%)	67	37	34	53	19	29		
SD	23	29	32	29	17	29		

Values in bold indicate guidelines which could be recommended for use in clinical practice.

## Data Availability

The data used to support the findings of this study are available from the corresponding author upon request.

## Abbreviations

AGREE: Appraisal of Guidelines for Research and Evaluation
CNO: Charcot neuro-osteoarthropathy
CPG: Clinical practice guideline
PRISMA-ScR: Preferred Reporting Items for Systematic reviews and Meta-Analyses extension for Scoping Reviews
SD: Standard deviation
IQR: Interquartile range

## Supporting Information

Supporting Information 1 Preferred Reporting Items for Systematic reviews and Meta-Analyses extension for Scoping Reviews (PRISMA-ScR) guideline checklist.

Supporting Information 2 Letter to D-Foot International members.

Supporting Information 3 Data extraction sheet template.

Supporting Information 4 AGREE-II scoring template.

Supporting Information 5 Eligible countries, how guideline was obtained, guideline name, and list of reviewers.
