# Supplementary material for: Review and Evaluation of European National Clinical Practice Guidelines for the Treatment and Management of Active Charcot Neuro-Osteoarthropathy in Diabetes Using the AGREE-II Tool Identifies an Absence of Evidence-Based Recommendations
Source: J Diabetes Res. 2024 Jun 10;2024:7533891. doi: 10.1155/2024/7533891 (PMC11186686; doi:10.1155/2024/7533891)
Supplement: Supporting Information 2 — Letter to D-Foot International members. [file 7533891.f2.pdf]

Charcot Foot Research Group
Diabetic Foot Study Group
DFSG Secretariat
c/o CAP Partner
Nordre Fasanvej 113, 2.
DK-2000 Frederiksberg
Denmark

Date:

Dear___________,

We are group of early career researchers, supported by five mentors, located across Europe who have come together to improve Diabetic Charcot Neuroarthropathy diagnosis and management. More information about our group can be found here.

Using a scoping review methodology, we aim to examine and evaluate the similarities, disparities and evidence level of current treatment and non-surgical management guidelines of acute CN within Europe. Results will be presented at this year's DFSG annual meetings in Bratislava, Slovakia.

We are currently collating guidelines for Charcot Foot, or where Charcot Foot is a component within the guideline, for analysis. To ensure we include all guidelines published within Europe, we would be delighted if you could send us the guidelines published within you region. This can be sent to dfsgcharcot@gmail.com.

Thank you for taking the time to read our letter, and many thanks in advance for your assistance.

King regards,

The DSFG Charcot Research Group
dfsgcharcot@gmail.com

Early Career Researchers
Dr Nichola Renwick, UK
Dr Catherine Gooday, UK
Dr Rasmus Bo Jansen
Ms Jennifer Pallin, IRL
Dr Aroa Tardáguila García
Dr Irene Sanz Corbalan
Dr Anastasios Tentolouris

Mentors
Dr Nina Petrova, UK
Dr Anna Korzon, PL
Dr Alexandra Jirkovska, CZ
Dr Armin Koller, DE
Prof Frances Game, UK
