# Supplementary material for: Review and Evaluation of European National Clinical Practice Guidelines for the Treatment and Management of Active Charcot Neuro-Osteoarthropathy in Diabetes Using the AGREE-II Tool Identifies an Absence of Evidence-Based Recommendations
Source: J Diabetes Res. 2024 Jun 10;2024:7533891. doi: 10.1155/2024/7533891 (PMC11186686; doi:10.1155/2024/7533891)
Supplement: Supporting Information 3 — Data extraction sheet template. [file 7533891.f3.pdf]

Baseline information					
Author	Link to Guideline	Year	Title	Country	Language

Method of Translation	Publishing Organization	Version	How was the guideline obtained?	Number of words on Charcot within the guideline	Notes

Guideline type / main area of focus		
Diabetes	Diabetic Foot	Surgical

Summary of the contents of the guideline					
			Management		
Definition	Diagnosis	Monitoring	Offloading	Pharmacological	Surgery

Remission	
Definition	Observation
