# Supplementary material for: Review and Evaluation of European National Clinical Practice Guidelines for the Treatment and Management of Active Charcot Neuro-Osteoarthropathy in Diabetes Using the AGREE-II Tool Identifies an Absence of Evidence-Based Recommendations
Source: J Diabetes Res. 2024 Jun 10;2024:7533891. doi: 10.1155/2024/7533891 (PMC11186686; doi:10.1155/2024/7533891)
Supplement: Supporting Information 4 — AGREE-II scoring template. [file 7533891.f4.pdf]

Domain 1: Scope and Purpose					
1. The overall objective(s) of the guideline is (are) specifically described		2. The health question(s) covered by the guideline is (are) specifically described		3. The population (patients, public, etc.) to whom the guideline is meant to apply is specifically described	
Rating	Comments	Rating	Comments	Rating	Comments

Domain 2: Stakeholder Involvement					
4. The guideline development group includes individuals from all relevant professional groups		5. The views and preferences of the target population (patients, public, etc.) have been sought		6. The target users of the guideline are clearly defined	
Rating	Comments	Rating	Comments	Rating	Comments

Domain 3: Rigours of Development					
7. Systematic methods were used to search for evidence		8. The criteria for selecting the evidence are clearly described		9. The strengths and limitations of the body of evidence are clearly described	
Rating	Comments	Rating	Comments	Rating	Comments

10. The methods for formulating the recommendations are clearly described		11. The health benefits, side effects, and risks have been considered in formulating the recommendations		12. There is an explicit link between the recommendations and the supporting evidence	
Rating	Comments	Rating	Comments	Rating	Comments

13. The guideline has been externally reviewed by experts prior to its publication		14. A procedure for updating the guideline is provided	
Rating	Comments	Rating	Comments

Domain 4: Clarity of Presentation					
15. The recommendations are specific and unambiguous		16. The different options for management of the condition or health issue are clearly presented		17. Key recommendations are easily identifiable	
Rating	Comments	Rating	Comments	Rating	Comments

Domain 5: Applicability					
18. The guideline describes facilitators and barriers to its application		19. The guideline provides advice and/or tools on how the recommendations can be put into practice		20. The potential resource implications of applying the recommendations have been considered	
Rating	Comments	Rating	Comments	Rating	Comments

21. The guideline presents monitoring and/or auditing criteria	
Rating	Comments

Domain 6: Editorial Independence			
22. The views of the funding body have not influenced the content of the guideline		23. Competing interests of guideline development group members have been recorded and addressed	
Rating	Comments	Rating	Comments

Overall Assessment	
1. Rate the overall quality of this guideline	2. I would recommend this guideline for use
