# Supplementary material for: Review and Evaluation of European National Clinical Practice Guidelines for the Treatment and Management of Active Charcot Neuro-Osteoarthropathy in Diabetes Using the AGREE-II Tool Identifies an Absence of Evidence-Based Recommendations
Source: J Diabetes Res. 2024 Jun 10;2024:7533891. doi: 10.1155/2024/7533891 (PMC11186686; doi:10.1155/2024/7533891)
Supplement: Supporting Information 5 — Eligible countries, how guideline was obtained, guideline name, and list of reviewers. [file 7533891.f5.pdf]

		Identified through								
	Country	Systematic search	Email contacts	Email response	Received guideline	Guideline met inclusion criteria	Confirmation of no guideline	Unable to obtain guideline	Guideline obtained	Reviewers
1	Albania	**X**	**X**	**n/a**	**X**			**X**		
2	Armenia	**X**	**X**	**n/a**	**X**			**X**		
3	Austria	**X**	**YES**	**YES**	**YES**	**YES**			Wochenschr WK, Lechleitner M, Abrahamian H, et al. leitlinien für die praxis Schlüsselwörter Diabetische Neuropathie · Diabeti-scher Fuß. Wien Klin Wochenschr. Published online 2019. doi:10.1007/s00508-019-1487-4	**CG, JP**
4	Azerbaijan	**X**	**X**	**n/a**	**X**			**X**		
5	Belarus	**X**	**X**	**n/a**	**X**			**X**		
6	Belgium	**X**	**YES**	**NO**	**X**			**X**		
7	Bulgaria	**X**	**YES**	**NO**	**X**			**X**		
8	Croatia	**X**	**X**	**n/a**	**X**					
9	Czechia	**X**	**YES**	**YES**	**YES**	**YES**			Czech Diabetological Society. DOPORUĆENÝ POSTUP PRO PREVENCI, DIAGNOSTIKU A TERAPII SYNDROMU DIABETICKÉ NOHY.; 2016.	**RBJ, AT**
10	Denmark	**X**	**YES**	**YES**	**YES**	**YES**			Danish Society of Endocrinology. Diabetisk Fodsygdom.; 2014. Accessed September 28, 2023. https://endocrinology.dk/nbv/diabetes-melitus/den-diabetiske-fod/	**AT, ATG**
11	England, Wales & Northern Ireland	**X**	**YES**	**YES**	**YES**	**YES**			NICE Clinical Guidelines. *Diabetic Foot Problems Prevention and Management*.; 2019. Accessed April 7, 2022. https://www.nice.org.uk/guidance/ng19	**RBJ, ISC**
12	Finland	**X**	**YES**	**YES**	**YES**	**YES**			The Finnish Medical Society Duodecim. Diabeetikon Jalkaongelmat.; 2021.	**ATG, RBJ**

13	France	X	YES	NO	X			X		
14	Georgia	X	X	n/a	X			X		
15	Germany	X	YES	YES	YES	YES			German Diabetes Association: Clinical Practice Guidelines. *Diabetic Foot Syndrome*.; 2021.	**JP, ISC**
16	Greece	X	YES	YES	YES	YES			Hellenic Diabetes Association. Κατευθυντήριες Οδηγίες Για Τον Σακχαρώδη Διαβήτη.; 2023. Accessed September 28, 2023. www.ede.gr/κατευθυντήριες-οδηγίες-εδε/	**ISC, ATG**
17	Hungary	X	X	n/a	X			X		
18	Iceland	X	YES	YES	X		X			
19	Ireland	X	YES	YES	X		X			
20	Italy	X	YES	NO	X			X		
21	Kazakhstan	X	YES	YES	YES	YES			Ministry of Health Republic of Kazakhstan. КЛИНИЧЕСКИЙ ПРОТОКОЛ ДИАГНОСТИКИ И ЛЕЧЕНИЯ СИНДРОМ ДИАБЕТИЧЕСКОЙ СТОПЫ.; 2019.	**AT, NR**
22	Kosovo	X	YES	NO	X			X		
23	Latvia	X	X	n/a	X			X		
24	Liechtenstein	X	X	n/a	X			X		
25	Lithuania	X	X	n/a	X			X		
26	Luxembourg	X	X	n/a	X			X		
27	Malta	X	YES	NO	X		X			
28	Moldova	X	X	n/a	X			X		
29	Montenegro	X	X	n/a	X			X		
30	Netherlands	X	YES	YES	YES	YES			Federatie Medisch Specialisten. *Diabetische Voet - Diagnostiek van Acute Charcot-Voet*.; 2021. Accessed September 28, 2023.	**NR, RBJ**
31	Northern Macedonia	X	X	n/a	X			X		
32	Norway	X	YES	NO	X			X		

33	Poland	YES	YES	YES	YES	YES			Diabetes Poland (Polish Diabetes Association). 2021 Guidelines on the Management of Patients with Diabetes: A Position of Diabetes Poland.; 2021.	**JP, CG**
34	Portugal	X	YES	YES	X		X			
35	Romania	X	YES	YES	YES	YES			Chirilă , Vlad, Radulian G. 7. Piciorul diabetic Ghid de management al diabetului zaharat. Published online 2021.	**JP, RBJ**
36	Russia	X	YES	YES	YES	YES			Российская ассоциация эндокринологов. КЛИНИЧЕСКИЕ РЕКОМЕНДАЦИИ ПО ДИАГНОСТИКЕ И ЛЕЧЕНИЮ СИНДРОМА ДИАБЕТИЧЕСКОЙ СТОПЫ.; 2015.	**AT, RBJ**
37	San Marino	X	X	X	X			X		
38	Scotland	X	YES	YES	YES	YES			Scottish Intercollegiate Guidelines Network. Management of Diabetes A National Clinical Guideline.; 2017. Accessed June 30, 2022. www.sign.ac.uk/assets/	**NR, CG**
39	Serbia & Herzegovina	X	YES	YES	YES	NO				
40	Slovakia	X	X	X	X			X		
41	Slovenia	X	YES	YES	YES	YES			Urbančič Rovan V, Lunder M, Ferjan S, Saletinger R, Lejko Zupanc T. 16. DIABETIČNA NOGA in Slovenske smernice za klinično obravnavo sladkorne bolezni tipa 2,. Published online 2022.	**ISC, RBJ**
42	Spain	X	YES	YES	YES	YES			Instituto Nacional de Gestión Sanitaria. Guía Para La Prevención y Cuidado Del Pie Del Paciente Diabético.; 2018.	**ATG, RBJ**
43	Sweden	X	YES	YES	YES	YES			Nationellt programområde endokrina sjukdomar. Personcentrerat och sammanhållet vårdförlopp Diabetes	**RBJ, JP**

									med hög risk för fotsår. Published online 2022.	
44	Switzerland	**X**	**YES**	**YES**	**YES**	**YES**			Peter-Riesch B, Czock A, Uçkay I. Swiss interdisciplinary guidance on good practices for acute and complicated diabetic foot syndromes. Swiss Med Wkly. 2021;151(45-46). doi:10.4414/SMW.2021.W30045	**AT, RBJ**
45	Turkey	**X**	**YES**	**YES**	**X**		**X**			
46	Ukraine	**X**	**YES**	**NO**	**X**			**X**		
